# Chromosomal-scale *de novo* genome assemblies of Cynomolgus Macaque and Common Marmoset

**DOI:** 10.1038/s41597-021-00935-6

**Published:** 2021-06-28

**Authors:** Vasanthan Jayakumar, Osamu Nishimura, Mitsutaka Kadota, Naoki Hirose, Hiromi Sano, Yasuhiro Murakawa, Yumiko Yamamoto, Masataka Nakaya, Tomoyuki Tsukiyama, Yasunari Seita, Shinichiro Nakamura, Jun Kawai, Erika Sasaki, Masatsugu Ema, Shigehiro Kuraku, Hideya Kawaji, Yasubumi Sakakibara

**Affiliations:** 1 Department of Biosciences and Informatics, Keio University, Yokohama, Kanagawa 223-8522 Japan; 2 Laboratory for Phyloinformatics, RIKEN Center for Biosystems Dynamics Research, Minatojimaminami-machi 2-2-3, Kobe, Hyogo, 650-0047 Japan; 3 RIKEN Center for Integrative Medical Science Preventive Medicine and Applied Genomics Unit, 1-7-22 Suehiro-cho, Tsurumi-ku, Yokohama, 230-0045 Japan; 4 Research Center for Genome & Medical Sciences, Tokyo Metropolitan Institute of Medical Science, 2-1-6 Kamikitazawa, Setagaya-ku, Tokyo, 156-8506 Japan; 5 RIKEN Center for Integrative Medical Sciences RIKEN-IFOM Joint Laboratory for Cancer Genomics, 1-7-22 Suehiro-cho, Tsurumi-ku, Yokohama, 230-0045 Japan; 6 RIKEN Preventive Medicine and Diagnosis Innovation Program, 1-7-22 Suehiro-cho, Tsurumi-ku, Yokohama, 230-0045 Japan; 7 Institute for the Advanced Study of Human Biology, Kyoto University, Yoshida-Konoe-cho, Sakyo-ku, Kyoto, 606-8501 Japan; 8 Department of Medical Systems Genomics, Graduate School of Medicine, Kyoto University, Yoshida-Konoe-cho, Sakyo-ku, Kyoto, 606-8501 Japan; 9 IFOM-the FIRC Institute of Molecular Oncology, Milan, Italy; 10 RIKEN Center for Integrative Medical Sciences Laboratory for Comprehensive Genomic Analysis, 1-7-22 Suehiro-cho, Tsurumi-ku, Yokohama, 230-0045 Japan; 11 Department of Stem Cells and Human Disease Models, Research Center for Animal Life Science, Shiga University of Medical Science, Shiga, 520-2192 Japan; 12 Institute for the Advanced Study of Human Biology (WPI-ASHBi), Kyoto University, Kyoto, 606-8501 Japan; 13 Central Institute for Experimental Animals, Department of Marmoset Biology and Medicine, Central Institute for Experimental Animals, 3-25-12, Tonomachi, Kawasaki-ku, Kawasaki, 210-0821 Japan

**Keywords:** Genome informatics, Biological techniques

## Abstract

Cynomolgus macaque (*Macaca fascicularis*) and common marmoset (*Callithrix jacchus*) have been widely used in human biomedical research. Long-standing primate genome assemblies used the human genome as a reference for ordering and orienting the assembled fragments into chromosomes. Here we performed *de novo* genome assembly of these two species without any human genome-based bias observed in the genome assemblies released earlier. We assembled PacBio long reads, and the resultant contigs were scaffolded with Hi-C data, which were further refined based on Hi-C contact maps and alternate *de novo* assemblies. The assemblies achieved scaffold N50 lengths of 149 Mb and 137 Mb for cynomolgus macaque and common marmoset, respectively. The high fidelity of our assembly is also ascertained by BAC-end concordance in common marmoset. Our assembly of cynomolgus macaque outperformed all the available assemblies of this species in terms of contiguity. The chromosome-scale genome assemblies produced in this study are valuable resources for non-human primate models and provide an important baseline in human biomedical research.

## Abstract (summary)

**Table:** 

Measurement(s)	sequence_assembly • genome • DNA
Technology Type(s)	sequence assembly process • DNA sequencing
Sample Characteristic - Organism	Macaca fascicularis • Callithrix jacchus

Machine-accessible metadata file describing the reported data: 10.6084/m9.figshare.14665314

## Background & Summary

Cynomolgus macaque (or crab-eating macaque, *Macaca fascicularis*) and common marmoset (*Callithrix jacchus*), belonging to old world monkey and new world monkey respectively, have been widely used in human biomedical research and drug developments with expectations that they recapitulate human physiology and pathology^1^. Their genomes, consisting of 42 and 46 chromosomes in diploids^2–4^, respectively, were assembled initially using first- and second-generation sequencing technologies. Short-read *de novo* assembly was not able to resolve complex repetitive genomic regions, and the resulting contigs tended to remain fragmentary. Techniques such as mate-pair sequencing were commonly used to join the contigs into longer scaffolds, albeit with sequence gaps in between. Long-standing non-human primate (NHP) genome assemblies released earlier used the human genome for ordering and orienting the assemblies into chromosomes, which prevents the observation of intrinsic structural differences between the primate genomes^5^. For example, a large inversion of around 20 Mb was observed in chromosome 16 of the earlier marmoset genome assembly, which should have been the result of the ‘humanization’ bias^6^.

Recent technological advancements allow us to obtain chromosome-scale assemblies without relying on existing genome assemblies, where such errors or bias can be avoided. Single-molecule long-read sequencing (Pacific Biosciences [PacBio] and Oxford Nanopore Technologies) have drastically increased the contiguity of assemblies, and chromatin contact profiling with Hi-C and other techniques such as optical mapping have paved the way to reconstructing chromosome-scale sequences. Taking advantage of these recent advancements, the genome sequences of some non-human primates (NHP) including gorilla, orangutan, and chimpanzee were largely improved^5,7^, followed by the ones for bonobo (Bioproject accession: PRJNA526933), and Northern white-cheeked gibbon (PRJNA369439). High-quality genome assemblies of old world monkeys were also recently reported, such as the ones for Rhesus macaque by three different research groups (PRJNA476474^8^, PRJNA509445^9^, and PRJNA514196^10^), the ones for olive baboon (PRJNA527874^11^), golden snub-nosed monkey (PRJNA524949^12^), and Francois’s langur (PRJNA488530^13^). We had also previously produced pseudo-chromosome assembly by using PacBio long-reads for common marmoset^6^, where ‘humanized’ sequences were still used as a reference.

In this study, we focus on cynomolgus macaque and common marmoset to establish a solid baseline for human biomedical research. We performed genome sequencing and *de novo* assembly for both species by using PacBio long-reads, along with Hi-C for chromosome-scale scaffolding through optimized Hi-C data acquisition^14^. We corrected misjoins of the scaffolds through examination of the Hi-C contact maps. We further investigated misjoins through cross-checking alternate contigs made from PacBio data and corrected them. Lastly, we assessed the quality of the resultant assemblies based on the contiguity of assembled sequences (N50), completeness of conserved protein-coding genes, and consistency to BAC clone sequences. The results indicate that our genome assemblies are of chromosomal-scale contiguity, with nearly complete coverage of gene space, and highly concordant to the conventional genomic resource obtained independently to our data set. Above all, for cynomolgus macaque, our genome assembly achieved the optimal quality in comparison with other resources available for this species.

## Methods

### Sample preparation, sequencing, and *de novo* assembly

**Fig. 1:**
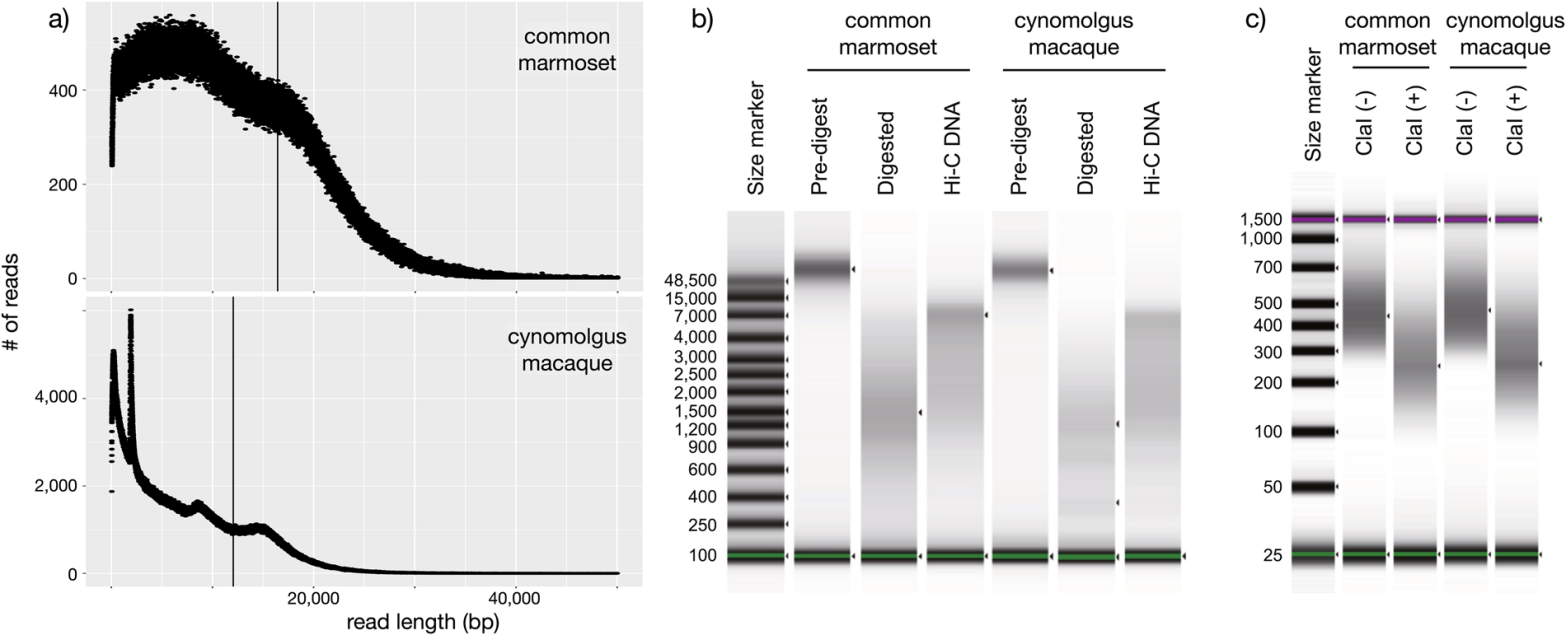
Quality assessment of the experiments. (**a**) Read length distribution of the common marmoset and cynomolgus macaque genomes, with a vertical line representing the N50 length. (**b**) Length distribution of the Hi-C DNA analyzed with Agilent TapeStation using the Genomic DNA ScreenTape. (**c**) Length distribution of the Hi-C library analyzed with Agilent TapeStation using the High Sensitivity D1000 ScreenTape.

A cynomolgus monkey was purchased by Shiga University of Medical Science from Shin Nippon Biomedical Laboratories, Ltd through Angkor Primates Center Inc in Kingdom of Cambodia. The identification number is CE1976F in Shiga University of Medical Science and K150090 in Shin Nippon Biomedical Laboratories, Ltd. We followed the Reporting *in Vivo* Experiments (ARRIVE) guidelines developed by the National Centre for the Replacement, Refinement & Reduction of Animals in Research (NC3Rs). All animal experimental procedures were approved by the Animal Care and Use Committee of Shiga University of Medical Science (approval number: 2017-10-2 (H1)). Genomic DNA (gDNA) was extracted from the kidney of the 6-year-8-month-old female cynomolgus macaque with MagAttract HMW DNA kit (48) [Qiagen 5067–5583; Cat No 67563] according to the manufacturer’s instruction. After measurement of concentration with Qubit3 - Qubit dsDNA (BR) [Thermo Fisher Scientific] and optical density (OD) with nanoDrop 2000 [Thermo Fisher Scientific], ethanol precipitation of the extracted gDNA was performed with DNA Clean & Concentrator-10 (25) [Zymo research] to condense it. Then, concentration and OD of the gDNA were again measured with NanoDrop 2000, Qubit3 - Qubit dsDNA (BR): the amount, 70.6 μg (307 ng/μL × 230 μL); 260/280, 1.87; 260/230, 2.28. Also, the successful extraction of long gDNA was confirmed by electrophoresis with TapeStation ScreenTape GenomicDNA on TapeStation [Agilent]. Using PacBio Sequel II, approximately 81x sequence data of the cynomolgus macaque was sequenced with a read N50 length of 12.05 kbp. For common marmoset, the 43x PacBio RSII read dataset from our earlier sequencing effort (PRJDB8242), was used to reassemble the genome (Fig. 1a; Table 1).

**Table 1:** Overview of the obtained sequences.

Species	PacBio Seqeuncing			iconHi-C		Note
	Sample	Sequencer	Read coverage	Sample	Read #	
Common marmoset	kidney (male)	RS II	43x	skeletal muscle (female)	408 M reads	PacBio reads obtained from Jayakumar, V. *et al*. 2020
Cynomolgus macaque	kidney (female)	Sequel II	81x	skeletal muscle (female)	377 M reads	The samples are obtained from the same individual

To assemble both the NHP genomes, we employed a series of assembly programs (supplementary fig. S1). Based on the performances from the earlier marmoset genome assembly study, we chose the assemblers Flye^15^, Redbean (wtdbg2)^16^, SMARTdenovo^17^, and miniasm^18,19^. After *de novo* assembly, all the assemblies were processed by PacBio’s polishing tool, arrow, to eliminate possible sequencing errors. After considering the results from multiple *de novo* assembly tools, Redbean was chosen as the base assembler, as it produced the most contiguous assemblies for both the common marmoset (contig N50 of 8.04 Mb; Table 2) and the cynomolgus macaque (contig N50 of 5.84 Mb) genomes (Table 3).

**Table 2:** Properties of the obtained and existing assemblies of common marmoset.

Assembly							
Name	calJac3 (Callithrix jacchus-3.2)	CJ2019*	mCalJac1.pat*	cj1700* (Callithrix_jacchus_cj1700_1.1)	Redbean assembly based on PacBio	Scaffolds based on iconHi-C	calJacRKC1912 (our final assembly; CJA1912RKC)
BioSample	GCA_000004665.1	GCA_009811775.1	GCA_011100535.1	GCA_009663435.2			GCA_013373975.1
BioProject	SAMN02981242	SAMD00169834	SAMN12368443	(SAMN12783337)			SAMD00217773
WGS project	PRJNA20401	PRJDB8242	PRJNA558087	PRJNA566173			PRJDB9375
RefSeq acc	ACFV01	BJKT01	JAALXR01	WJHW01			BLSI01
Synonym	GCF_000004665.1			GCF_009663435.1			
Release date	22-Jan-2010	23-Dec-2019	10-Mar-2020	22-May-2020			03-Jun-2020
**Sequence length statistics**							
Number of scaffolds	14,205	65	336	964	5,008	4,303	1,872
Total length of scaffolds (Gb)	2.91	2.79	2.68	2.90	2.81	2.81	2.81
Maximum scaffold length (Mb)	210.4	213.3	217.0	218.0	75.4	208.0	208.5
N50 scaffold length (Mb)	132.2	143.9	137.0	137.7	8.0	134.9	132.3
Number of scaffolds > 10 Mb	23	23	22	23	65	23	23
Proportion of scaffolds > 10 Mb (%)	94.9	99.5	98.9	97.38	45.6	97.0	97.3
Number of gaps (‘N’ tracts)	187,214	1,771	788	380	0	2,908	1,846
Number of contigs >10 Mb	0	54	82	79	65	65	74
Maximum contig length (Mb)	0.4	46.0	84.3	124.0	75.4	75.4	160.6
N50 contig length (Mb)	0.0	6.4	14.7	25.2	8.0	7.4	24.8
**Completeness of gene space**^**+**^							
Complete orthologs (%)	92.70	93.13	92.70	93.56	90.13	90.99	92.27
Complete + fragmented orthologs (%)	97.42	97.85	97.42	97.85	97.42	97.85	97.42
Missing orthologs (%)	2.58	2.15	2.58	2.15	2.58	2.15	2.58
Average number of copies per ortholog	1.17	1.15	1.15	1.17	1.17	1.16	1.15
Detected multi-copy orthologs (%)	12.96	12.44	12.04	13.30	14.29	13.21	12.09
**BAC end alignment**							
	71.35/88.98	86.44/93.84	83.68/89.91	88.16/94.27	82.85/93.12	84.90/93.10	85.81/93.30

*The current reference assemblies and the other ones deposited recently.

^+^The completeness assessment was performed by the computational pipeline CEGMA and the reference gene set CVG.

**Table 3:** Properties of the obtained and existing assemblies of cynomolgus macaque.

Assembly						
Name	macFas5*	Macaca_fascicularis_6.0*	mfascicularis_v7_1*	Redbean assembly based on PacBio	Scaffolds based on iconHi-C	macFasRKS1912 (our final assembly; MFA1912RKS)
GenBank acc	GCA_000364345.1	GCA_011100615.1	GCA_903231565.1			GCA_012559485.1
BioSample	SAMN00811240	SAMN14146612	SAMEA6828341			SAMD00204697
BioProject	PRJNA20409	PRJNA607781	PRJEB37977			PRJDB9269
WGS project	AQIA01	JAANEP01	CAFBRT01			BLPH01
RefSeq acc	GCF_000364345.1					
Synonym	macFas5					
Release date	20-May-2013	09-Mar-2020	03-Jun-2020			26-Mar-2020
**Sequence length statistics**						
Number of scaffolds	7,601	936	184	2,467	1,247	400
Total length of scaffolds (Gb)	2.95	2.91	2.51	2.80	2.80	2.80
Maximum scaffold length (Mb)	227.6	223.6	208.3	34.4	217.7	222.1
N50 scaffold length (Mb)	152.8	150.4	137.7	5.8	148.9	149.9
Number of scaffolds >10 Mb	21	22	20	57	23	21
Proportion of scaffolds >10 Mb (%)	97.5	97.7	99.5	29.9	99.4	99.7
Number of gaps (‘N’ tracts)	80,474	560	7,308	0	1,752	604
Number of contigs >10 Mb	0	91	0	57	55	79
Maximum contig length (Mb)	0.8	95.2	6.3	34.4	34.4	93.0
N50 contig length (Mb)	0.1	21.3	0.9	5.8	5.6	26.3
**Completeness of gene space**^**+**^						
Complete orthologs (%)	91.42	90.13	78.54	91.42	93.13	93.13
Complete + fragmented orthologs (%)	98.71	97.00	91.85	98.28	99.14	99.14
Missing orthologs (%)	1.29	3.00	8.15	1.72	0.86	0.86
Average number of copies per ortholog	1.08	1.09	1.08	1.07	1.06	1.05
Detected multi-copy orthologs (%)	7.04	7.62	7.65	5.63	4.61	4.61
**BAC end alignment**						
	—	—	—	—	—	—

*The current reference assemblies and the other ones deposited recently.

^+^The completeness assessment was performed by the computational pipeline CEGMA and the reference gene set CVG.

### Hi-C data acquisition and scaffolding

Hi-C libraries were constructed with the restriction enzyme DpnII following the iconHi-C protocol^14^, using the skeletal muscle tissues of an adult female marmoset and a cynomolgus macaque that were kept frozen in −80 °C after dissection and snap-freezing in liquid nitrogen. Fixed tissue materials containing 2 μg of DNA were used for the preparation of Hi-C DNA by *in situ* restriction digestion and ligation. Library preparation was performed using 1 μg of Hi-C DNA with 5 cycles of PCR for the marmoset and 6 cycles of PCR for the cynomolgus macaque. Quality control of the Hi-C DNA and the Hi-C library was performed as described in the iconHi-C protocol^14^. Quality control of the Hi-C DNA showed an expected pattern of a shift in size – shortening after digestion and elongation after ligation, indicating successful preparation of the Hi-C DNA (Fig. 1b). Quality control of the Hi-C library by restriction digestion confirmed the existence of expected ligation junction sequences inside the library molecules at a high proportion and successful generation of the library (Fig. 1c).

Sequencing to obtain Hi-C reads was performed on an Illumina HiSeq X in paired-ends with 151 cycles. The obtained reads were processed using Trim Galore to remove low-quality regions and adapter sequences. Post-sequencing quality control of the Hi-C libraries was performed as described previously^14^. Hi-C read statistics obtained by HiC-Pro^20^, using one million subsampled read pairs from the large scale sequencing data mapped to the PacBio contigs, confirmed high quality of the libraries while showing a high proportion of valid interaction read pairs, a low proportion of invalid ligation products (dangling end pairs), and a low proportion of contiguous restriction fragments (re-ligation pairs) (Supplementary table S1). Approximately 408 and 377 million Hi-C reads for the marmoset and the cynomolgus macaque were mapped to the Redbean contig sequences of the common marmoset and the cynomolgus macaque respectively using Juicer^21^. With the Juicer output files, Hi-C scaffolding was performed using 3d-dna^22^. Inversions and misjoins in the assemblies that occurred during the Hi-C scaffolding process were corrected by using Juicebox based on the frequency of Hi-C contacts^23^.

**Fig. 2:**
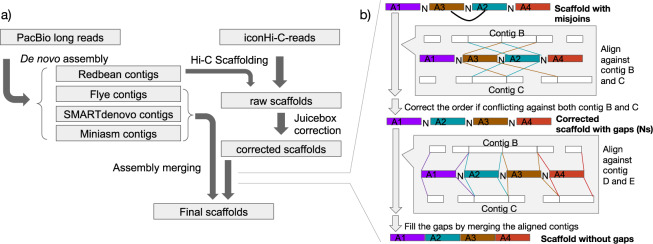
Computational steps of genome assembly. (**a**) *De novo* assembly and Hi-C scaffolding workflow. (**b**) Misjoin correction and gap-filling using contigs from genome assemblies using alternate assembly tools.

**Fig. 3:**
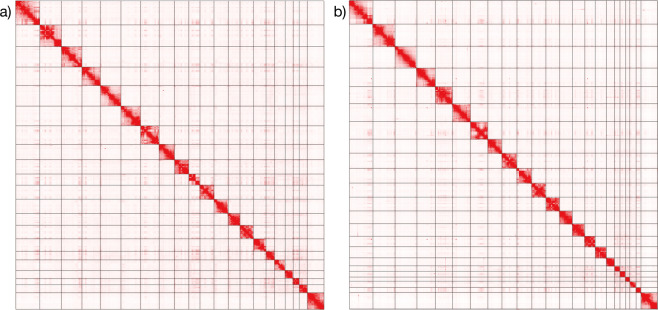
Hi-C contact map on the final assemblies. Hi-C contact maps of the pseudo-chromosome assemblies of (**a**) common marmoset and (**b**) cynomolgus macaque.

As a result of the genome assembly workflow involving de novo assembly followed by Hi-C scaffolding (Fig. 2a), the N50 lengths increased from approximately 8 to 135 Mb for the marmoset (Table 2) and from 5.8 to 149 Mb for the cynomolgus macaque (Table 3), and the number of scaffolds longer than 10 Mb presumed to be at the chromosome level became close to the actual number of chromosomes (Fig. 3). For the cynomolgus macaque, two pairs of the scaffold sequences longer than 10 Mb were merged into two pseudo-chromosomes, referred to as chromosomes 2 and 8 later, based on alignment concordance with the 3d-dna scaffold sequences of alternate assemblies, as well as the previous reference sequence.

### Misjoin detection and gap-filling

All the alternate assemblies were also independently scaffolded using 3d-dna. These were later aligned against the Hi-C scaffolds from Redbean assembly to observe whether any misjoins were introduced by Hi-C scaffolding. Because the contiguity profiles of the assemblies are different across the employed assemblers, contigs which are broken into two or more sequences in one assembly could have been assembled into a single contiguous piece in another assembler (Supplementary fig. S1). This contiguity information from alternate assemblies was used to identify misjoins in the scaffold sequences from the Redbean assembly (Fig. 2b). When the alignments of the alternate assemblies against Redbean scaffolds were visualized using D-genies dot plots^24^, occasionally the scaffolds did not exhibit consistency in chromosomal structure among the 3d-dna scaffolds. These indicated the presence of misjoins introduced by Hi-C scaffolding (Supplementary fig. S2).

In case that such discrepancies in contig orders were observed in the alignments, a two-step check was performed, A) whether gaps are present in the Redbean scaffolds, and B) whether at least two of the alternate assemblies are consistent in those regions and discrepant against the Redbean scaffolds. If the above case is true, instead of re-ordering the misjoined scaffolds, we replaced the alignment block with one of the alternate assemblies making it a hybrid assembly. A similar procedure was also performed for gap-filling in regions without misjoins also. When a gap-containing region in Redbean scaffolds aligned to a gap-free region of contigs from alternate assemblies, those gap-containing regions were replaced with the corresponding blocks from alternate assemblies (Fig. 2b). When two or more alternate assemblies can be used to fill the gaps, one of them was randomly chosen to fill the gaps, followed by additional two rounds of polishing using arrow. This gap-filling procedure filled 1,147 gaps of length 573.94 kb in cynomolgus macaque, and 1,061 gaps of length 522.16 kb in common marmoset respectively.

## Data Records

The N50 length of the contigs increased from 8.04 Mb to 24.82 Mb, and from 5.84 Mb to 26.27 Mb for the final assemblies of common marmoset and cynomolgus macaque genomes, respectively. The final genome assemblies displayed chromosome-sized sequences, designated here as ‘pseudo-chromosomes’, which exhibited scaffold N50 lengths of 132.27 Mb and 149.88 Mb, for the cynomolgus macaque and the common marmoset genomes, respectively (Table 2; Table 3).

The raw genome sequence data of the cynomolgus macaque are deposited at the DDBJ under the accession, DRA009584^25^. The Hi-C raw sequence data are deposited under the accessions, DRA009641^26^, and DRA009987^27^, for the cynomolgus macaque and the common marmoset genomes, respectively. The assembled genome sequences are deposited under the accessions, BLPH01000001-BLPH01000400^28^, and BLSI01000001-BLSI01001872^29^, for the cynomolgus macaque and the common marmoset genomes, respectively.

## Technical Validation

### Gene space completeness assessment

The web server gVolante^30^, in which two established pipelines CEGMA^31^ and BUSCO^32^ are implemented, was used to assess the sequence length distributions and gene space completeness in a uniform environment. The latter was based on the coverage of one-to-one reference orthologues with the ortholog search pipeline CEGMA and the gene set CVG that is specifically optimized to assess vertebrate genome sequences^33^. CEGMA identified pre-selected conserved protein-coding genes by using a combination of tblastn, and GeneWise for sequence alignment^34,35^, and GeneID for gene prediction^36^, in addition to HMMER3 for further homology search^37^, which are used to assess the completeness of the genome assemblies. The analysis of gene space completeness revealed a smaller number of missing CVG genes for both species, in comparison with the scores for the genome assemblies released earlier (Table 2; Table 3).

### BAC-end alignment

BAC-end read pairs of the common marmoset from our earlier sequencing effort^4^, were aligned against the constructed assembly using bowtie2^38^. After the misjoin detection and gap-filling procedure, the number of concordantly aligned read pairs increased by a count of 684, while the number of discordantly aligned read pairs decreased by a count of 401 (Table 2).

### Comparison of common marmoset assembly against previous assemblies

**Fig. 4:**
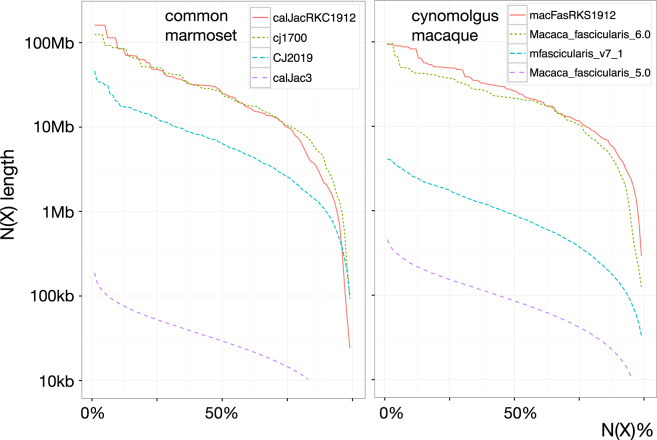
Contiguity plots of the existing and obtained assemblies. N(X) plots comparing the contiguity profiles of existing and obtained genome assemblies of (**a**) common marmoset and (**b**) cynomolgus macaque. The contig lengths of MacFas_Jun2011 are well below 10 kb and did not fit into the scale of the plotted graph, and hence MacFas_Jun2011 was omitted from the figure.

For the common marmoset, NCBI hosts nine genome assemblies (https://www.ncbi.nlm.nih.gov/assembly/organism/9483/latest/), with cj1700 being the representative reference genome. The assembled genome in this study has a total scaffold length of 2.81 Gb with a contig N50 length of 24.82 Mb, in comparison to the total scaffold length of 2.79 Gb with the contig N50 length of 6.38 Mb from our earlier effort (CJ2019). Our newly assembled contigs have slightly lesser contiguity than the recently submitted assembly cj1700 which has a total scaffold length of 2.9 Gb with a contig N50 length of 26.62 Mb (Fig. 4a). Notably, our assembly is 23 Mb longer than cj1700 after excluding gap regions, and more than half of the sequence gaps in the pseudo-chromosomal sequences of the cj1700 assembly were identified as contiguous regions in our assembly. These results imply that these assemblies are similarly useful and, in some difficult regions, complementary to each other (Supplementary table S2).

### Comparison of cynomolgus macaque assembly against previous assemblies

NCBI hosts five cynomolgus macaque genome assemblies (https://www.ncbi.nlm.nih.gov/assembly/organism/9541/latest/), with the macFas5 genome assembly being the representative reference genome (Table 3). Three of the assemblies including the representative genome have contig N50 lengths shorter than 100 kbp. In contrast, the recently submitted Macaca_Fascicularis_6.0 assembly had produced a much larger contig N50 length of 21.34 Mb. It has a total scaffold length 2.91 Gb, which is smaller than that of the representative reference genome’s 2.95 Gb. The assembly from our present study has a contig N50 length of 26.27 Mb and a total scaffold length 2.91 Gb. It is 65 Mb shorter than Macaca_Fascularis_6.0 even after excluding gap regions, and the ideal total length of the assembly is not yet conclusive. Notably, our assembly outperformed all the assemblies in terms of contiguity (Fig. 4b) and its coverage of orthologous genes in our assembly achieved the highest ratio, 93%, whereas the one in Macaca_Fascicularis_6.0 was 90% (Table 3). It indicates that the assembly produced here outperformed the rest not only in terms of contiguity, but also in base accuracy.

### Comparison against other non-human primate genome assemblies

The contiguity of the two genomes assembled in this study was compared against other non-human primate genomes such as those of Rhesus macaque, Francois langur, chimpanzee, gorilla, orangutan, white-cheeked gibbon, golden snub-nosed monkey, bonobo, and olive baboon. Although there were three different sub-species genome assemblies for Rhesus macaque, we herein considered only the best assembly out of them in terms of contiguity. In comparison to the other non-primate genome assemblies, it was evident that the common marmoset and cynomolgus macaque assemblies produced the second and the third-best contiguities, with the Rhesus macaque genome assembly^8^, being the best among all in terms of contiguity (Supplementary fig. S3).

## Supplementary information

Supplementary Information

## Data Availability

The versions of the tools used and their parameters are described as follows. Flye v2.3.6-release: flye --pacbio-raw -g 2.9 g Redbean (Wtdbg2) v2.3: wtdbg2 -x rsII -g 2.7 g -L 5000 wtpoa-cns -i ctg.lay.gz For cynomolgus macaque, rsII was replaced by sq. SMARTdenovo (git commit 3d9c22e25bdf4caf6c08ea1acb41ee58e52f61a8): Default parameters with consensus generation. Minimap2 v2.10-r761 and miniasm (git commit 17d5bd12290e0e8a48a5df5afaeaef4d171aa133): minimap2 -x ava-pb | gzip -1 > m.paf.gz miniasm -f reads.fastq m.paf.gz > tigs.gfa For cynomolgus macaque, only reads longer than 10 kb were considered for the assembly. Hi-C scaffolding: Trim Galore v0.4.5: --paired --phred33 -e 0.1 -q 30 HiC-Pro v2.11.1: default parameters Juicer v20180805: default parameters 3d-dna v20180929: -m haploid -i 5000 -r 2 Juicebox v1.3.6 Polishing: Pbmm2 v0.12.0: pbmm2 align assembly.referenceset.xml reads.subreadset.xml aln.alignmentset.xml --sort -j 18 -J 18 -m 5000 M Variant Caller v2.3.2: The consensus sequence was split into 50 parts and the arrow algorithm was executed using default parameters. Arrow polishing was iteratively executed twice for flye and SMARTdenovo assemblies, and thrice for miniasm and redbean assemblies. Validation: gVolante v1.2.1: (CEGMA v2.5, CVG v10042017, NCBI BLAST v2.2.31, GeneWise v2.2.3-rc7, GeneID v1.4, HMMER v3.1b2) cegma.mod.pl --interlen 100000 --boundaries 10000 --ext --protein CVG/CVGs.fa --hmm_profiles CVG/hmm_profiles --cutoff_file CVG/profiles_CVG_cutoff.tbl --complete_file CVG/completeness_CVG_cutoff.tbl
