# Supplementary material for: Chromosomal-scale de novo genome assemblies of Cynomolgus Macaque and Common Marmoset
Source: Sci Data. 2021 Jun 28;8:159. doi: 10.1038/s41597-021-00935-6 (PMC8239027; doi:10.1038/s41597-021-00935-6)
Supplement: Supplementary file 1 — Supplementary Information [file 41597_2021_935_MOESM1_ESM.pdf]

Supplementary Information

Chromosomal-scale *de novo* genome assemblies of Cynomolgus Macaque and Common Marmoset

Vasanthan Jayakumar, Osamu Nishimura, Mitsutaka Kadota, Naoki Hirose, Hiromi Sano, Yasuhiro Murakawa, Yumiko Yamamoto, Masataka Nakaya, Tomoyuki Tsukiyama, Yasunari Seita, Shinichiro Nakamura, Jun Kawai, Erika Sasaki, Masatsugu Ema, Shigehiro Kuraku, Hideya Kawaji, & Yasubumi Sakakibara.

Summary

The manuscript describes the construction of chromosome-scale genome assemblies of two non-human primates, cynomolgus macaque and common marmoset. This document contains supplementary figures S1,S2, S3 and supplementary tables S1, and S2.

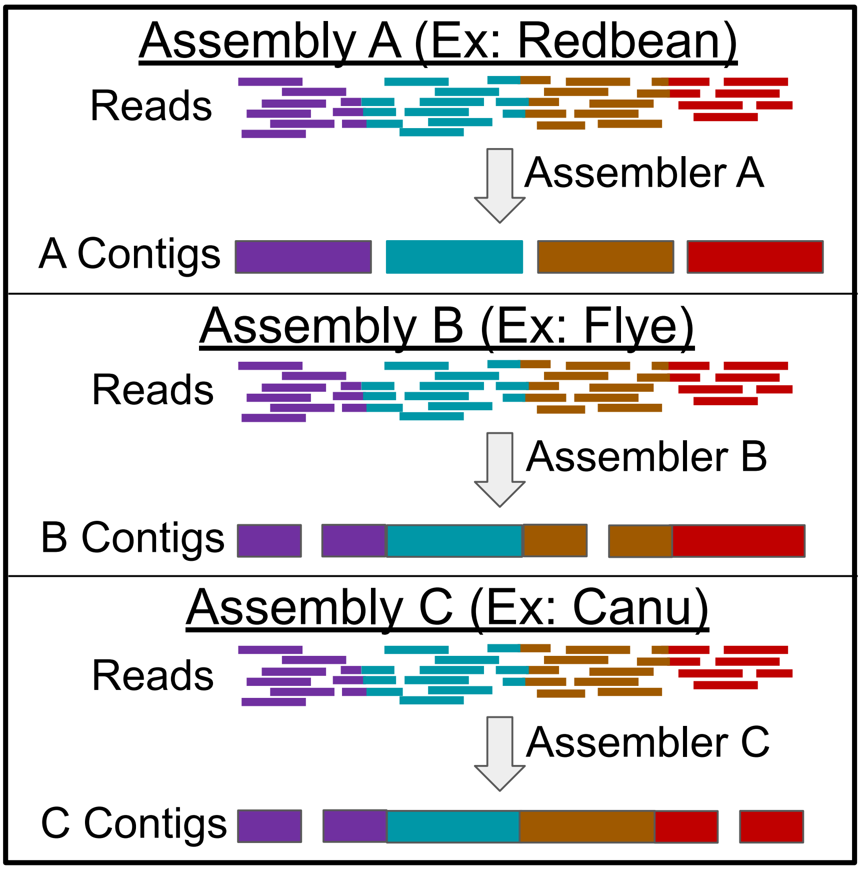

Supplementary figure S1. *De novo* assembly from multiple assemblers
Multiple *de novo* assembly tools employed to obtain different contiguity profiles.

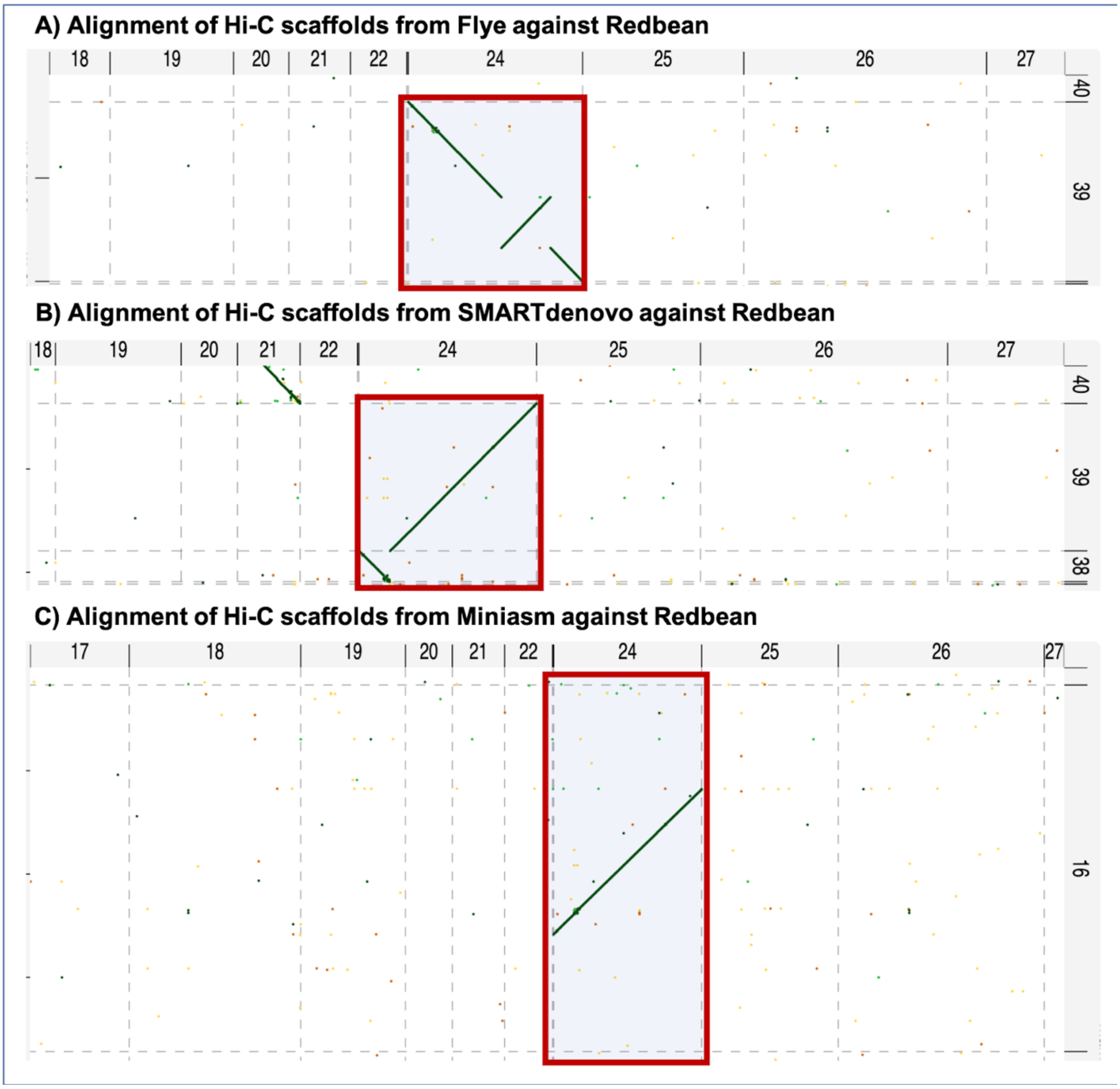

Supplementary figure S2. Misjoins in Hi-C scaffolding

Hi-C scaffolding of genome assemblies produced from different assembly tools revealed misjoins.

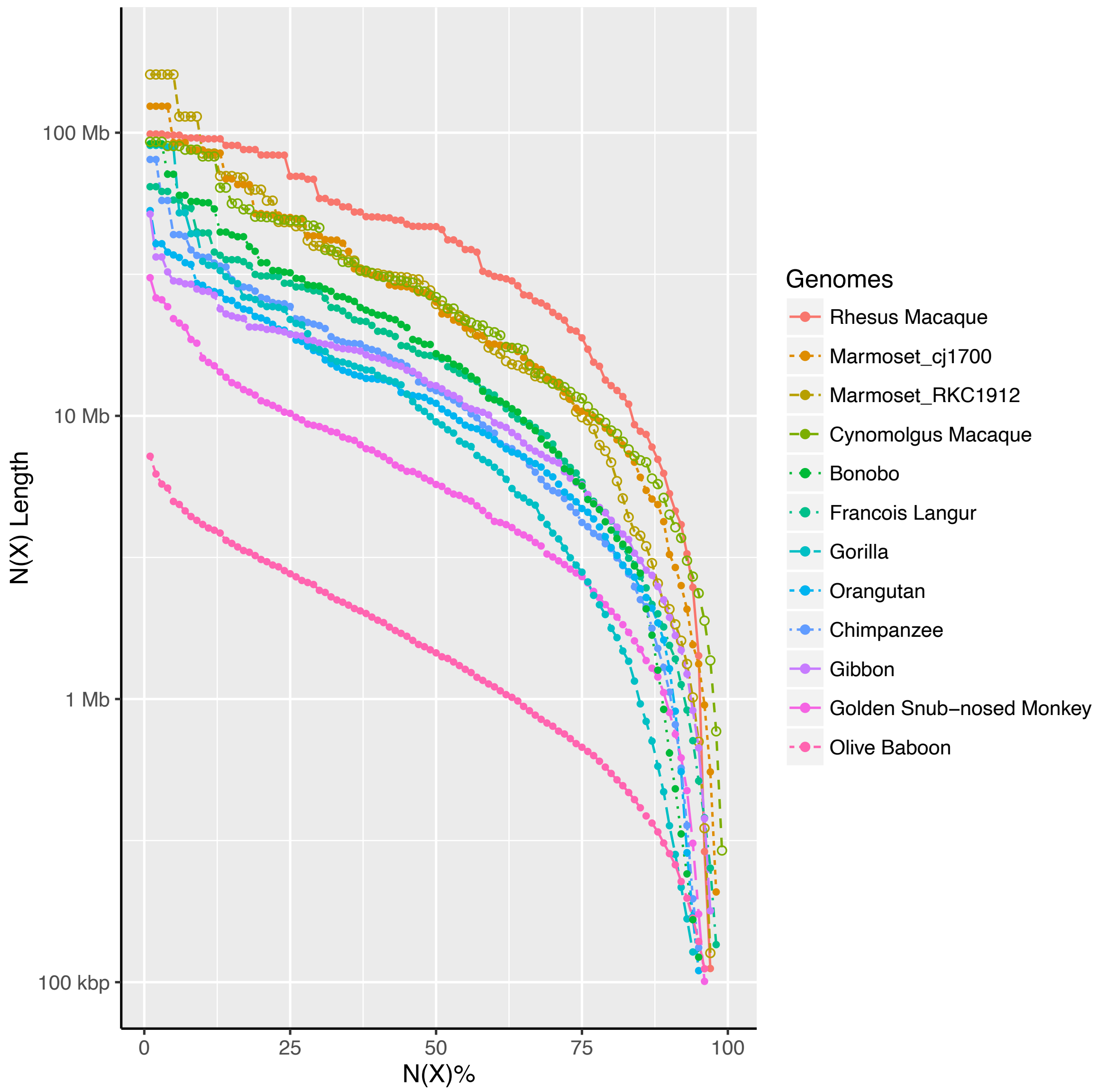

Supplementary figure S3. Contiguity plots of non-primate genome assemblies
N(X) plots comparing the contiguity profiles of publicly available non-human primate genome assemblies.

Supplementary table S1: Proportion of the Hi-C libraries

Read pair category	Common marmoset	Cynomolgus macaque
Unique paired alignments	75.2%	67.1%
Valid Hi-C pairs	72.9%	65.4%
Dangling end pairs	1.0%	0.9%
Re-ligation pairs	1.2%	0.8%
Self circle pairs	0.1%	0.1%
Single-end pairs	0.0%	0.0%
Filtered pairs	0.0%	0.0%
Dumped pairs	0.0%	0.0%
Unmapped pairs	0.6%	1.2%
Low quality pairs	0.0%	0.0%
Multiple pairs alignments	17.4%	21.0%
Pairs with singleton	6.7%	10.6%
Low quality singleton	0.0%	0.0%
Unique singleton alignments	0.0%	0.0%
Multiple singleton alignments	0.0%	0.0%

Supplementary table S2. Gaps in cj1700 genome assembly filled by calJacRKC1912

FASTA ID of cj1700	Total gaps (Ns)	Gaps filled by calJacRKC1912	% of gaps filled
NC_048383.1	34	13	38.24
NC_048384.1	25	22	88.00
NC_048385.1	8	5	62.50
NC_048386.1	20	5	25.00
NC_048387.1	21	7	33.33
NC_048388.1	13	9	69.23
NC_048389.1	11	7	63.64
NC_048390.1	4	3	75.00
NC_048391.1	14	7	50.00
NC_048392.1	14	7	50.00
NC_048393.1	11	10	90.91
NC_048394.1	16	4	25.00
NC_048395.1	18	13	72.22
NC_048396.1	18	8	44.44
NC_048397.1	11	4	36.36
NC_048398.1	8	1	12.50
NC_048399.1	6	4	66.67
NC_048400.1	12	4	33.33
NC_048401.1	7	2	28.57
NC_048402.1	6	5	83.33
NC_048403.1	11	8	72.73
NC_048404.1	26	17	65.38
NC_048405.1	32	9	28.13
All pseudo-chromosomes	346	174	50.29
